# Self-organization, free energy minimization, and optimal grip on a field of affordances

**DOI:** 10.3389/fnhum.2014.00599

**Published:** 2014-08-12

**Authors:** Jelle Bruineberg, Erik Rietveld

**Affiliations:** ^1^Amsterdam Brain and Cognition, University of AmsterdamAmsterdam, Netherlands; ^2^Department of Philosophy, Institute for Logic, Language and Computation, University of AmsterdamAmsterdam, Netherlands; ^3^Department of Neurology, Max Planck Institute for Human Cognitive and Brain SciencesLeipzig, Germany; ^4^Department of Psychiatry, Academic Medical Center, University of AmsterdamAmsterdam, Netherlands

**Keywords:** affordances, self-organization, metastability, optimal grip, Merleau-Ponty, neurodynamics, Free Energy Principle, landscape of affordances

## Abstract

In this paper, we set out to develop a theoretical and conceptual framework for the new field of Radical Embodied Cognitive Neuroscience. This framework should be able to integrate insights from several relevant disciplines: theory on embodied cognition, ecological psychology, phenomenology, dynamical systems theory, and neurodynamics. We suggest that the main task of *Radical Embodied Cognitive Neuroscience* is to investigate the phenomenon of skilled intentionality from the perspective of the self-organization of the brain-body-environment system, while doing justice to the phenomenology of skilled action. In previous work, we have characterized skilled intentionality as the organism's tendency toward an optimal grip on multiple relevant affordances simultaneously. Affordances are possibilities for action provided by the environment. In the first part of this paper, we introduce the notion of skilled intentionality and the phenomenon of responsiveness to a field of relevant affordances. Second, we use Friston's work on neurodynamics, but embed a very minimal version of his Free Energy Principle in the ecological niche of the animal. Thus amended, this principle is helpful for understanding the embeddedness of neurodynamics within the dynamics of the system “brain-body-landscape of affordances.” Next, we show how we can use this adjusted principle to understand the neurodynamics of selective openness to the environment: interacting action-readiness patterns at multiple timescales contribute to the organism's selective openness to relevant affordances. In the final part of the paper, we emphasize the important role of metastable dynamics in both the brain and the brain-body-environment system for adequate affordance-responsiveness. We exemplify our integrative approach by presenting research on the impact of Deep Brain Stimulation on affordance responsiveness of OCD patients.

## Introduction

This *Frontiers* special issue on *Radical Embodied Cognitive Neuroscience* invites researchers to re-imagine cognitive neuroscience in terms of (radical) embodied cognitive science. Radical Embodiment is the view that cognition ought to be understood primarily in terms of the embodied agent—environment dynamics. Neural dynamics can only be studied while taking into account the larger brain-body-environment dynamics (Chemero, 2009). Besides highlighting the dynamical aspects of cognition, embodied cognitive science has also highlighted the importance of phenomenology and ecological psychology for studying cognition. In this paper, we develop a theoretical and conceptual framework that aims to integrate some of the various fields of study that come together in a *Radical Embodied Cognitive Neuroscience*: neurodynamics, ecological psychology, phenomenology, self-organization and dynamical systems theory.

The starting point of this paper is the question how skilled agents interact with their environment and can tend toward improvement of their situation. In particular, we are interested in how, in a particular context, skilled agents are selectively responsive to only some of the many available “affordances” or possibilities for action offered by their environment (Gibson, 1979; Chemero, 2003). In order to understand this, phenomenology suggests that we need to complement Gibson's original theory of affordances with an understanding of the attracting or soliciting character of affordances in relation to an agent in a particular situation (Rietveld, 2008a; Withagen et al., 2012). We think that the main task of *Radical Embodied Cognitive Neuroscience* is to explain how the changing world and the dynamics of the agent's state mesh together in a way that makes adequate action possible, while simultaneously doing justice to the phenomenology of skilled action. In this paper we theoretically and conceptually develop a framework for investigating this. Although the phenomenon of skilled activity is relevant for both humans and non-human animals (Ingold, 2000), we will focus on human beings in this paper. Also, we will limit ourselves to agents who have already acquired their skills. So we will not focus on developing, learning, fine-tuning, and modifying skills nor on the evolutionary history of skilled behaviors, although these topics raise important open issues as well.

In the first part of this paper, we focus on the phenomenon of selective affordance-responsiveness because that is an ecologically valid way to characterize the dynamics of the system “skilled agent—environment.” In the second part of the paper, we show how theoretical neuroscience can help to understand selective affordance-responsiveness. First, we introduce the framework of self-organization in order to bring the necessary conceptual tools to the table. Second, we focus on how neurodynamics is embedded in the dynamics of the broader brain-body-environment system. We present the Free Energy Principle (FEP) as a promising framework to understand this embeddedness but, inspired by Anderson and Chemero (2013), interpret it in a more minimal way than has previously been done. Furthermore, we show how we can use this adjusted framework to understand the neurodynamics of selective openness to affordances. Next, we argue for a situated understanding of the FEP in which the self-organizing brain is understood as coordinating action-readiness patterns to deal with relevant affordances. In the final part of the paper, we illustrate the plausibility of our conceptual framework by showing how it is able to integrate findings on metastable dynamics in the brain-body-environment system, and how it is able to shine new light on the effects of Deep Brain Stimulation (DBS) on treatment resistant obsessive-compulsive disorder (OCD).

## Skilled intentionality and optimal grip on a field of affordances

Affordance-responsiveness is a central feature of everyday skillful activity of both humans and non-human animals (Rietveld, 2012a). Affordances are possibilities for action provided to an animal by the substances, surfaces, objects, and other living creatures that surround it (Gibson, 1979; Reed, 1996; Heft, 2001; Chemero, 2003, 2009; Silva et al., 2013). Affordances can be defined as relations between aspects of the material environment and abilities available in a form of life (Rietveld and Kiverstein, under review; cf. Chemero, 2003).

Up till now in the field of Embodied Embedded Cognition affordances have typically been understood as motor possibilities the environment offers to a creature, such as reaching, grasping, sitting, walking etc. Developing a Wittgensteinian account of affordances, we (Rietveld and Kiverstein, under review) have argued that for creatures that inhabit a resourceful social and cultural environment as we do, the possibilities for action the environment offers are far richer: the affordances on offer in the landscape of affordances available in our form of life are related to the whole spectrum of abilities available in our human socio-cultural practices (cf. Heft, 2001). Both unreflective action in everyday life and episodes of what are traditionally called “higher” cognition are forms of *skilled* interaction with the environment and can be understood in terms of responsiveness to affordances (Rietveld, 2008c, 2013).

Based on a careful reading of Gibson, we have recently shown (Rietveld and Kiverstein, under review), that contrary to what many think, it is not affordances but the ecological *niche* for a kind of animal with a particular way of life that forms the cornerstone of Gibson's ideas. Our notion of the landscape of available affordances was introduced to do justice to this *primacy of the niche*, which is present independently of perception by a particular individual (See Box 1). The astonishing richness of the landscape of available affordance in our niche hinges on the fact that both relata of affordances, both the sociomaterial environment and the reservoir of abilities in our socio-cultural practices, manifest an enormous variety.

Box 1 **Terminology of skilled intentionality.**

***AFFORDANCE:*** A possibility for action provided by the environment to an animal.

***SOLICITATION:*** An affordance that stands out as relevant for a particular animal in a specific situation.

***SKILLED INTENTIONALITY:*** The kind of intentionality an individual exhibits when acting skillfully in a familiar situation (see Text for elaboration). We characterize skilled intentionality as the tendency toward an optimal grip on a field of affordances.

***TENDENCY TOWARD AN OPTIMAL GRIP:*** The tendency of a skilled individual to be moved to improve its grip on the situation by responding to solicitations.

***LANDSCAPE OF AFFORDANCES:*** The affordances available in an ecological niche. In our human form of life, these are related to the whole spectrum of abilities available in our socio-cultural practices.

***FIELD OF AFFORDANCES:*** The affordances that stand out as relevant for a particular individual in a particular situation; i.e., the multiplicity of affordances that solicit the individual.

This enormous richness raises the question how an organism can be responsive to only the *relevant* affordances in a given situation. Phenomenologically, some of the affordances around us do not leave us cold but move us. In earlier work (Rietveld, 2008a) we have suggested that an affordance can “invite” or “solicit” behavior dependent on the current concerns of the organism and the situation it is in (Withagen et al., 2012). The metaphor of a *field* is useful here: some affordances stand out more than others. Some are experienced as soliciting immediately, others are experienced as soliciting on the horizon and still others are completely ignored (only the latter do in fact leave us cold). We can distinguish between an affordance, i.e., a possibility for action available in our form of life at a certain location, and a solicitation. A solicitation is an affordance that stands out as relevant in a specific situation lived by an animal. “Action readiness” (Frijda, 1986, 2007) is a useful notion here, because it is a phenomenon in between overt action and ability. A solicitation is the (pre-reflective) experiential equivalent of a bodily action readiness: the readiness of the affordance-related ability (Rietveld, 2008a).

Much of our everyday interactions with the environment, such as riding a bike through a city, moving toward an appropriate distance from other people in an elevator, or ordering a cup of coffee in a bar, can be described as skillful activities. In previous work, we have introduced the notion of *skilled intentionality* as the tendency toward an optimal grip on a situation by being selectively responsive to available affordances (Rietveld, 2008c, 2012a, 2013). The tendency toward an optimal grip^1^ is a primarily phenomenological notion that signifies the way a skilled individual acts in a familiar environment in order to improve its grip on the situation. What is central to this notion, is that the individual experiences the situation in terms of a deviation of an optimum. As Merleau-Ponty puts it:

For each object, as for each picture in an art gallery, there is an optimum distance from which it requires to be seen, a direction viewed from which it vouchsafes most of itself: at a shorter or greater distance we have merely a perception blurred through excess or deficiency. We therefore tend toward the maximum of visibility, and seek a better focus as with a microscope (Merleau-Ponty, 2002/1945, p. 352).

Importantly, during those episodes of skilled activity, the skilled individual does not have an explicit goal in mind, but rather is solicited by the environment in such a way as to improve her grip on the situation. Phenomenologically, this deviation of an optimum can be described as an experienced tension to be reduced. In the case of a skilled individual, which is what we focus on in this paper, tending toward grip is the equivalent of having an action readiness for dealing adequately with an affordance; one is responsive to, or poised to act adequately on an affordance.

We suggest that the tendency toward an optimal grip on the situation is a basic concern of living organisms and is a central feature of our everyday skillful dealings with our environment. It shapes the person's selective openness to the landscape of available affordances so that certain affordances “stand out” as relevant and the individual can unreflectively improve his or her situation by simply being responsive to this structured field of relevant affordances (Rietveld, 2008c, 2012a,b). For instance, when entering a crowded elevator, we stand at an appropriate distance from the other people.

It is this phenomenon of the tendency toward an optimal grip and especially how theories from the fields of self-organization and theoretical neuroscience can contribute to an understanding of context-sensitive selective openness to relevant affordances that is the central topic of this paper.

The specific structure of the field of affordances of a particular individual is dependent on the current concerns and abilities of that organism and the current situation. The structure of the field of affordances changes when either the landscape of affordances changes (i.e., when the sociomaterial environment changes or when the abilities available in a form of life change), or when the concerns of the individual change. If a rabbit eats the only carrot available in a certain place, it changes the layout of the (locally present) landscape of affordances. However, as the landscape of affordances changes and the individual's interest in eating diminishes, new possibilities for action show up. Once the carrot has been eaten, the rabbit hole might solicit sleeping, or a place a bit further away might solicit exploring (cf. Dreyfus, 2007).

**Figure 1:**
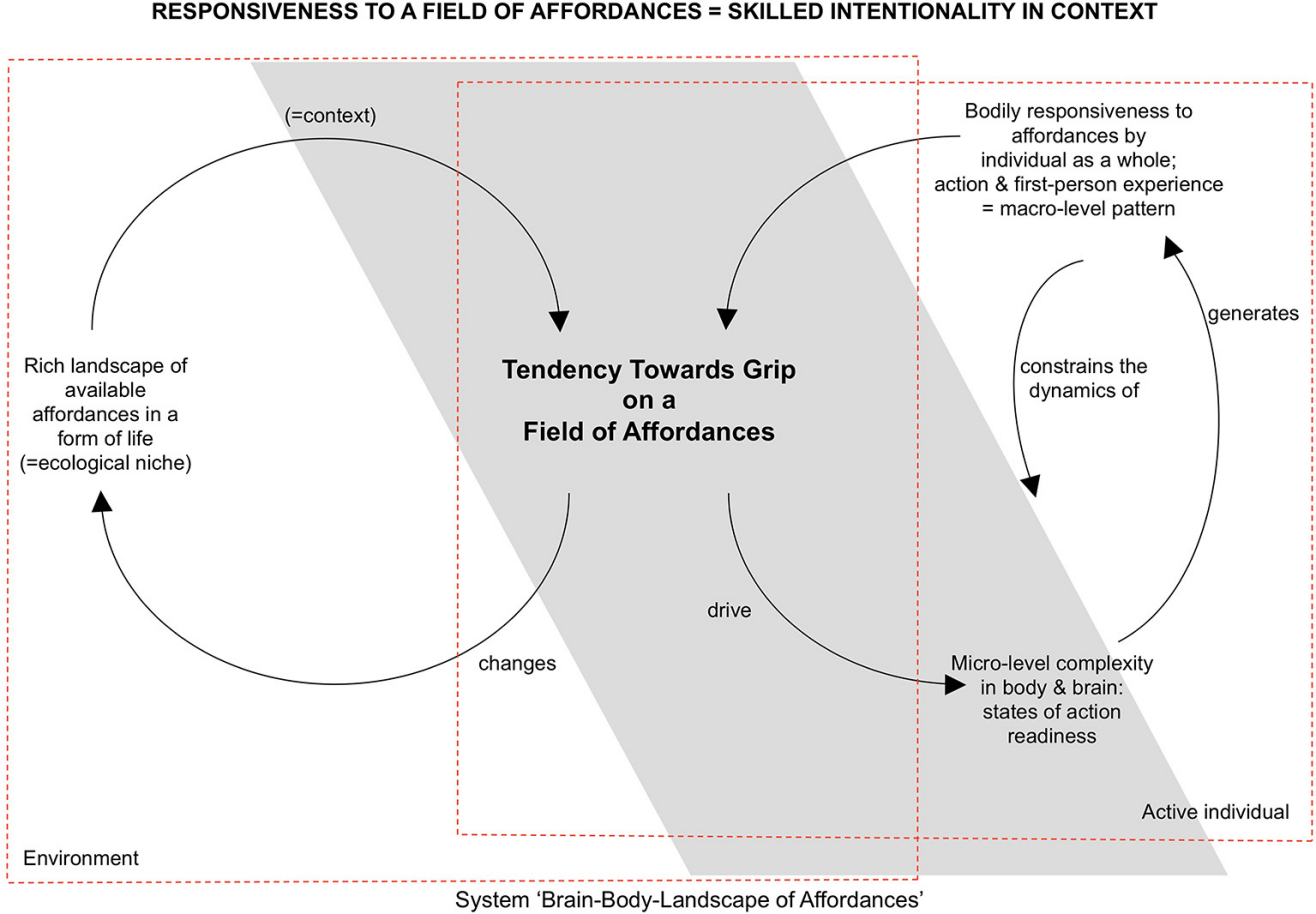
**Sketch of the conceptual framework to be refined**. Through skilled intentionality one gets a grip on a field of affordances (Rietveld, 2013) (inspired by Chemero, 2003, 2009; Dreyfus, 2007; Thompson, 2007, 2011; Tschacher and Haken, 2007; Rietveld, 2008a,b, 2012a,b).

Changes in the field of affordances can also originate in the environment. For the eating rabbit, a sound in the bushes might change the field in such a way that the carrot does not solicit eating anymore, but now the rabbit hole solicits hiding. An important part of skilled intentionality is therefore not only being skillfully responsive to one affordance, but also being open to changes in the context and adequately engaging with these affordances (see also Section Toward a Radical Embodied Cognitive Neuroscience on metastability). The tendency toward an optimal grip on a field of affordances is the result of a dynamic interplay between the landscape of affordances and the current state of the organism. On the side of the organism, states of action readiness interact in order to bring about selective openness to a landscape of affordances (see Figure 1). We will return to the processes of self-organization and neurodynamics contributing to selective openness in the subsequent sections of the paper. One aspect of the answer to the question of how individuals can get a grip on the multiplicity of affordances available already becomes clear from looking at the structure of the landscape of affordances.

### The structure of the landscape of affordances

The concept of a “*landscape* of affordances” aims to capture the interrelatedness of the available affordances. Affordances are not encountered as a set of separate possibilities for action, but rather as a *nested* structure of interrelated affordances^2^. In the case of the form of life of enculturated human beings, this structure can be very complex. It is only against the background of socio-cultural practices, places and institutions that the affordances here in my office are intelligible. The affordances of places (libraries, restaurants, etc.) typically constrain behavior over a longer timescale, while the affordances of objects nested in such a place, say the door to the library's reading room, typically constrain behavior on a shorter timescale^3^. Such place-affordances (the affordances of say, university libraries, railway stations, supermarkets, swimming pools or restaurants) are the contexts in which many of our activities unfold (Kiverstein and Rietveld, 2012; cf. Heft, 2001). Which affordances are relevant depends on the “behavior setting” (Barker, 1968; Heft, 2001): the possibility of calling a waiter is relevant in a restaurant but not when we are in a supermarket. Being in a restaurant constrains or pre-structures which affordances are relevant to me. In order to be responsive to the appropriate affordances of a situation (e.g., calling out a waiter in a restaurant), one needs to be well attuned to the current context (one needs to have the ability to deal and be ready to deal with restaurants and waiters). In sum, we suggest that responsiveness to a place-affordance, which is a nest of affordances, generates an action readiness that makes the individual selectively open to the landscape of affordances. As such this responsiveness pre-structures the relevance of locally available affordances in a way that allows the individual to have a grip on the rich landscape of affordances in which she is situated.

The nestedness of the landscape of affordances thus helps the organism to gain a grip on multiple relevant affordances simultaneously. The challenge for the organism is to, in a particular situation, be selectively open to only the relevant affordances. In the remainder of this paper we seek to find out how theoretical neuroscience and dynamical cognitive neuroscience contribute to understanding such self-organized relevance sensitivity.

## Self-organization

One of the developments relevant for an understanding of the mechanisms that contribute to selective affordance-responsiveness is an improved understanding of self-organizing systems. Especially, we are interested in self-organizing systems that are able to actively influence their interactions with the environment in order to adapt to and induce environmental changes, i.e., so called homeokinetic or self-serving systems (Iberall, 1977; Turvey and Carello, 2012). The theory of self-organization is particularly suitable for the framework of affordance-responsiveness developed here, because in both of these theoretical frameworks, it is the reduction of a tension or gradient that is the central motivation for an action: it is the environment that is the driving force for an action for an organism in a particular situation. We will first present the familiar Bénard effect as an example of how self-organizing patterns can be functional with respect to their environment and subsequently describe how the theory of self-organization can improve our understanding of affordance-responsiveness.

Self-organizing systems are initially disordered systems where global order can arise under the influence of the system's own dynamics. This is typically the case when a control parameter reaches a critical value upon which new forms of organization become possible for the system. Within the self-organizing range, the behavior of the system is low dimensional, i.e., it can be quantified by a small amount of order parameters that describe the macroscopic patterns in the system (See Box 2). Classical examples from the literature stem from diverse fields such as treatments of the Bénard cell in non-equilibrium fluid dynamics (Bénard, 1900; Bishop, 2008), the laser in optics (Haken, 2004) and coordination dynamics in cognitive science (Haken et al., 1985).

Box 2 **Terminology of complex and dynamical systems^4^.**

***STATE SPACE:*** The space defined by the set of all possible states a system could ever be in.

***TRAJECTORY (PATH):*** A set of positions in the state space through which the system might pass successively. The behavior of the system is often described by trajectories through the state space.

***ATTRACTOR:*** A point of state space to which the system will tend when in the surrounding region.

***TOPOLOGY (ATTRACTOR LANDSCAPE):*** The layout of attractors in the state space.

***CONTROL PARAMETER:*** Some parameter of a system whose continuous quantitative change leads to a non-continuous, qualitative change in the attractor landscape.

***ORDER PARAMETER:*** Some parameter of a system that summarizes the behavior of the system's components.

***CIRCULAR CAUSALITY:*** The mutually constraining relationship between the microscopic and macroscopic elements of a complex system: the order parameters emerge out of the microscopic dynamics, while the order parameters themselves constrain or enslave the microscopic dynamics.

***SECOND CIRCULARITY:*** The mutually constraining relationship between one or more control parameters in the environment and a self-organizing system. The system self-organizes in order to reduce the control parameter(s) that gives(s) rise to its self-organization.

***(CENTRAL) PATTERN GENERATOR:*** A dynamical system producing rhythmic patterned activity potentially modulated by feedback mechanisms.

***METASTABILITY:*** A property of coupled dynamical systems in which over time the system's tendency to integrate and segregate coexist.

### Rayleigh–Bénard convection

The Rayleigh–Bénard effect is empirically, theoretically and philosophically the most well studied non-linear self-organizing system. The phenomenon occurs when a layer of fluid is heated from below. Cold water is denser (hence heavier) than warm water, so the temperature difference creates a buoyancy force. When the temperature difference is small, the viscosity of the fluid counteracts the buoyancy force and the system will dissipate energy through heat conduction. When the temperature gradient passes a critical value, the buoyancy force overcomes the viscosity (more potential energy is brought in the system than can be dissipated through heat conduction) and the system becomes globally unstable. This leads to convection patterns in the shape of parallel cylinders (so called convection or Bénard rolls).

In the formalization of the Bénard effect, the temperature difference between the top and the bottom of the fluid is considered a control parameter. The macroscopic state of the system (conduction or convection) is a function of the control parameter. Furthermore, in the self-organizing regime, the system can be described and determined by only a few variables, the so-called order parameters. The relation between the order parameters and the microscopic components (the single molecules of the liquid, e.g., water molecules) is a peculiar one: the order parameters constrain the trajectories of the parts, but the parts also generate the order parameters. The relationship between parts (the microscopic) and whole (the macroscopic) is one of mutual constraints or, to use Tschacher and Haken's philosophically somewhat problematic term, *circular causality* (Tschacher and Haken, 2007).

### Gradient reduction and second circularity

How can the theory of self-organization help us to understand the mechanisms of the tendency toward an optimal grip in human beings? There is a second fact about self-organization in the Bénard system. The self-organization has an impact on the environment as well. The self-organization reduces the very temperature gradient that gives rise to it: it is the temperature difference that enables the convection, but the convection reduces the temperature difference. It is due to this so called second *circularity*, that self-organized patterns are functional with respect to their environment, that is to say: the patterns are *geared toward the reduction of the environmental gradients*^5^ on the system. Crucially, the *function* of self-organized pattern formation, according to Tschacher and Haken (2007), is to adapt to environmental constraints and realize dissipation of the gradients.

It is these two circularities that we find in affordance responsiveness as well. On the one hand, solicitations move the organism in a particular direction; on the other hand leads the responsiveness to the solicitation to a reorganization of the field of affordances, which makes new solicitations stand out. We therefore propose to think of relevant^6^ affordances as gradients that drive the dynamics of the system and in return are consumed by it.

There is, however, an important difference between a Bénard system and a system like the brain-body-environment system: in the Bénard effect and most other standard examples of self-organization, there is only one control parameter working on the system. For our purpose of understanding the mechanisms of optimal grip in the case of human beings, it is important to consider the case of multiple control parameters, because generally there are multiple relevant affordances in any particular situation of an individual^7^.

### Self-organization and living systems

There is another significant dissimilarity between systems like the Bénard system and systems like the affordance-responsive organism. In the case of non-living systems, as in the Bénard system, the self-organizing pattern disappears if the external control parameter decreases below a threshold. For example, if the temperature difference reaches below the critical value, the organized patterns disappear. Living systems have to be able to actively interact with the gradients that affect their self-organization. One could then say that the gradient is not *given by*, but *obtained from* the environment (Iberall, 1977; Turvey and Carello, 2012). In the first case, systems are *served* by the environment, while in the second case, systems are *self-serving* or homeokinetic^8^. These latter systems can internally generate forces to counteract the effect of physical gradients on the system, and move through their material environment to avoid harmful gradients and find new ones [this is what Turvey and Carello (2012, p.11) call “proto-foraging” behavior]. Crucially, through this capacity, the system is able to (within limits) influence the gradients that affect it and hence maintain its own self-organization (Kugler and Turvey, 1988; Turvey and Carello, 2012). In the hypothetical case of a *living* Bénard cell, this would amount to a layer of fluid being able to heat or cool itself, or to move through a temperature landscape in the environment in order to regulate its self-organizing patterns.

What is interesting about Tschacher and Haken's (2007) proposal is the conceptual link between gradients and affordances. They do emphasize that the reduction of gradients can also occur when more gradients work on a system, but in their (2007) account, the nature of these gradients and their structure remains undeveloped. The perspective we have sketched advances Tschacher and Haken's account of affordances in three ways. First, we distinguish conceptually between affordances and solicitations (Rietveld, 2008a; cf. Rietveld, 2008b; Withagen et al., 2012). Second, we show that each affordance is embedded in a landscape of affordances of a given form of life, which includes socio-cultural practices in our human form of life. The embeddedness in this landscape is crucial for adequate anticipation of the organism in its environment. It is only when we are attuned to the specific context—including place-affordances—that we can adequately be responsive to relevant solicitations that are in line with our concerns. Third, at the level of the individual as a whole we connect the reduction of gradients with the tendency toward an optimal grip on a concrete situation.

Our formulation of affordance-responsiveness in terms of self-organization does not yet address the problem of context-sensitive *selective* openness to affordances, which, as we have suggested in the introduction and earlier work (Kiverstein and Rietveld, 2012), should be the central topic of Radical Embodied Cognitive Neuroscience. The theories of self-organization and synergetics (Haken, 1983) provide the framework in which to investigate this important problem. In the upcoming sections of this paper we explore how a complex system like the brain can be selectively sensitive to only *some* environmental gradients/affordances.

## Anticipation and selective openness

In recent years, there has been growing interest in the application of ideas from statistical physics, machine learning and complex and dynamical systems theory to the brain (see for instance Freeman, 1987, 2000; Friston, 2006; Tognoli and Kelso, 2014). What these approaches have in common is their appreciation of the brain as an intrinsically active and unstable self-organizing system. In part thanks to these authors, progress has been made in how the self-organization of the brain can be functional with respect to the larger brain-body-environment dynamics (see also Freeman, 2000; Dreyfus, 2007). We think that this perspective (neurodynamics embedded in brain-body-environment dynamics) is the natural starting point to develop a Radical Embodied Cognitive Neuroscience.

One promising proposal to couple brain, body and environment is Karl Friston's FEP (Friston, 2010)^9^. According to the FEP, any self-organizing system that remains within physiological bounds in its interactions with a changing environment (and hence resist a natural tendency to disorder), can only frequent a limited amount of physical states. This can be given a mathematical interpretation in the sense that the probability distribution of the organism's states must have low entropy (i.e., there is a high probability that a system is in one of a relatively small number of states). This long term imperative to constrain the entropy of its states translates into a short term imperative to suppress surprisal^10^ (see Box 3). Importantly, surprisal can not be suppressed directly, since it depends on the expected range of states over time. The information theoretic quantity of free energy (not to be confused with the homologous concept from thermodynamics)^11^ is an upper bound on surprisal such that when an organism minimizes free energy, it is implicitly minimizing surprisal (Friston, 2011).

Box 3 **Information theory and the anticipating brain^12^.**

***SURPRISAL:*** A measure for the unexpectedness of an event expressed in terms of the negative log-probability of the event outcome.

***FREE ENERGY:*** An information theoretic measure that is an upper bound on the surprisal of some data, given a generative model.

***PREDICTION ERROR:*** The difference between anticipated and actual sensory input. Under simplifying assumptions, Free Energy equals the sum of prediction errors.

Importantly, free energy can be evaluated, because it is a function of the organism's sensory states and the organism's internal dynamics (called a generative model). Roughly, free energy is a measure for the “*dis-attunedness*” of the internal dynamics and the environmental dynamics. For example, it is low when the sensory states are anticipated, and high when they are not. The FEP says that minimizing free energy is a necessary and sufficient condition for self-organizing adaptive systems to maintain a robust brain-body-environment system and hence, remain within physiological bounds.

In the active inference formulation (Friston, 2010,2013b) of the FEP, free energy can be minimized on short time scales by making the environment conform to the internal dynamics (“action”) or by making the internal dynamics conform to the environmental dynamics (“perception”). There is an important similarity between Tschacher and Haken's framework of self-organization and Friston's FEP: what they call circular causality and second circularity map onto what Friston calls “perception” and “action,” respectively. It is through these two circularities that organism and environment are coupled.

The FEP in itself makes no claims about the mechanisms underlying free energy minimization. It is supposed to be a necessary requirement for any adaptive self-organizing system that is able to resist the tendency to disorder. When it comes to organisms with developed nervous systems, the FEP offers a rich and sophisticated set of tools in order to gain a better understanding of how free energy can be minimized. Given some simplifying assumptions (cf. Marreiros et al., 2009) the brain dynamics can be modeled using variational Bayesian methods and hierarchical predictive-coding. However, to avoid misunderstandings, it is important to distinguish between the imperative (i.e., minimizing free energy) and the mechanisms by which the organism obeys that imperative. As Friston himself notes: “The Bayesian brain and predictive-coding are […] seen as a consequence of […] this fundamental imperative [of free energy minimization.]” (Friston, 2013a, pp. 212–213). Free Energy minimization is thus the primary notion and we wish to foreground that, rather than the Bayesian and the predictive-coding framework^13^.

The FEP implies a deep connection between the dynamics of the brain-body-environment system and the neurodynamics. What is crucial, for the organism, is that it anticipates the kind of interactions with the environment that lead to an adequate outcome (such as having food, or avoiding a passing car). The function of the generative model is therefore not to provide the agent with a representation of the dynamical structure of the environment *per se*, but rather to steer its interactions with its environment in such a way that a *robust* brain-body-environment system is maintained. The internal dynamics, Friston's generative model, can not be understood apart from its functioning within the integrated brain-body-econiche system.

To illustrate this point, note that Friston himself states, somewhat provocatively, that: “each […] agent *embodies* an optimal model^14^ of its econiche” (Friston, 2011). Furthermore, Friston states that:

“[A]n agent does not *have* a model of its world—it *is* a model. In other words, the form, structure, and states of our embodied brains do not contain a model of the sensorium—they *are* that model. […] But what does this mean practically? It means that every aspect of our brain can be predicted from our environment” (Friston, 2013a, p. 213).

For Friston, the niche implies the structure of the organism. Now, for our argument, we do not need to subscribe to this last claim in the fullest sense, but it shows the radical potential of the FEP.

In general we think the FEP is a step forward in understanding the relation between environmental dynamics and neurodynamics. It is an attractive framework because we think it is able to formalize the tendency toward an optimal grip in terms of the dynamical coupling between brain dynamics and the dynamics of the whole brain-body-environment system, or more specific: of the whole system “brain-body-landscape of affordances.” Within the framework of the FEP the tendency toward an optimal grip could be seen as a consequence of the continuous minimization of free energy through perception and action at the level of the organism as a whole: *the attunement of the internal dynamics and external dynamics*.

However, we worry that along with the welcome mathematical sophistication comes a vocabulary that is mathematically convenient, but philosophically problematic (Anderson and Chemero, 2013). For instance, within philosophy and cognitive science the notion of “inference” is traditionally understood in terms of arriving at a propositional statement based on some premises or observations. Within the Free Energy framework, the notion of “inference” is much more minimal and does not involve any propositions: any dynamical system A coupled with another B can be said to “infer” the “hidden cause” of its “input” (the dynamics of B) when it reliably covaries with the dynamics of B and it is robust to the noise inherent in the coupling. [For a presentation of this minimal notion of inference, see Friston (2012b, 2013c)]. This is important, because it suggests that the apparent tension between radical embodiment and the FEP is at least to some extent terminological^15^.

To summarize, the FEP dictates that in order to maintain a robust brain-body-environment system, an organism can and needs to continuously minimize the prediction error or discrepancy (formalized in terms of free energy) between its internal dynamics and the dynamics of the larger system. The organism does not need to have a model *of* its niche, but rather the claim is that the structure of the niche is reflected in the structure of the skilled embodied organism. We will argue that the internal dynamics should be understood in terms of affordance-related action-readiness patterns. The notion of an econiche is not developed any further in Friston's work up to now, but we will come back to the relation between an organism's niche (made up of a landscape of affordances) and the internal dynamics in the Section on Situating the Anticipating Brain.

So far we have focused on integrating our theoretical framework of skilled intentionality with the theoretical framework of the FEP. The integration of these two frameworks now places us in a position to look at the neurodynamics of selective affordance-responsiveness under the FEP. It is here that the theory of self-organization, introduced in the previous section of this paper becomes important again.

## The neurodynamics of selective openness

In this section we will present a neurodynamical approach that is able to account for selective-responsiveness to affordances within the adjusted framework of the FEP. Within the Free Energy framework, selective responsiveness is brought about by pattern generators that make both sensory (exteroceptive) and motor (proprioceptive) predictions (Friston, 2012a)^16^. Pattern generators are well known through the work of Randall Beer on robot locomotion (Beer and Chiel, 1993). They are systems that are capable of producing rhythmic or sequential patterns and can be modulated by sensory feedback. Beer uses coupled pattern generators with sensory feedback to build distributed control circuits for robot locomotion. The dynamics of a pattern generator is modulated and constrained by both its sensory feedback and the dynamics of the other pattern generators.

Kiebel et al. (2009) show that by coupling pattern generators evolving at different timescales, one can create a dynamical system (a generative model in the sense introduced in the last section) that is capable of swiftly interacting with a complex dynamical environment. The pattern generator evolving at longer timescales serves as a control parameter that shapes the attractor at which the lower-level dynamics unfold. The specific kinds of pattern generators they use are so called stable heteroclinic channels (Rabinovich et al., 2008). These are defined as a sequence of metastable (saddle) points with transients in between^17^. When these stable heteroclinic channels are coupled in a temporal hierarchy, the ensuing dynamics never reaches a fixed stable point, but continuously follows a trajectory through state space (Kiebel et al., 2009). This trajectory is continuously modulated through sensory feedback (prediction errors). Some prediction errors can be accommodated for on the lower level, leaving the slower-evolving patterns intact (for instance when synchronizing to an external rhythm), while other prediction errors, can induce or destroy the pattern generators at a longer timescales as well (such as when the beat of the music changes dramatically).

This is important for understanding how the selective openness helps to make, in the particular situation, the distinction between the relevant affordance(s) and other affordances; between the one(s) to be responded to here and now and the ones that leave the organism cold. The generation of an adequate action-readiness rests upon precise sensory feedback that feeds into a dynamical system (generative model) that is shaped by the organism's previous interactions with the environment. The system will settle on a pattern that explains away most of the prediction error (i.e., the system tends toward a particular attractor). On slower time-scales this amounts to “action selection”, while on the faster timescales the action is specified: prediction errors influence the attractors that make more specific sensorimotor predictions (“action specification”). Both action selection and action specification depend on sensitivity to small disturbances that is, deviations from anticipations generated by pattern generators (Cisek, 2007; Cisek and Kalaska, 2010).

The fact that stable heteroclinic channels implement metastable attractor dynamics is crucial for understanding the flexibility of selective openness to affordances. Kelso (2012) describes metastability as the outcome of two competing tendencies: the tendency of the components to couple together and the tendency to express their independent behavior. In this metastable regime, the system is poised at the edge of instability, a kind of dynamic stability that allows the system to maintain “a balance in the readiness of the system to transit between multiple attractors” (Davids et al., 2012., p. 119). While being skillfully engaged with a specific task, it is important that we can be affected by affordances on the horizon of our field and rapidly switch to another kind of adequate activity when something in the environment changes. Metastable dynamics are important for understanding the brain, because metastability is a prerequisite for a system to be able to effortlessly switch between different patterns. We will see that metastability plays an important role as well in the brain-body-environment dynamics of skilled agents, in the Section: Toward a Radical Embodied Cognitive Neuroscience.

In Friston's picture, the elicitation of an action-readiness-pattern triggers a cascade of spatiotemporal dynamics in the brain modulated by sensory input that aids anticipation on the interactions with the environment. In ballroom dancing for instance, the first measures of music will afford either dancing tango or waltz. The elicitation of the tango-dancing-pattern will trigger an attractor-manifold that governs the sensorimotor coordination between me, my dance partner and the music: this action-readiness pattern will make certain action possibilities solicit more to me than others. On a more fine-grained level, small cues by the dance partner and subtle variations in the rhythm in the music further specify my action-readiness. Only if I am well attuned to the context (the situation) and thus metastably poised for several relevant activities I could do next, can small cues in the environment lead to very different positions in state space and hence to flexible responsiveness to (very) different solicitations. That is, only when I am able to rapidly accommodate the small deviations from my anticipations (in Friston's terms: the ability to explain away prediction errors through perception and action) can I engage skillfully with a complex environment.

Within our adjusted version of the FEP, a solicitation is a gradient/prediction error that, through action, can be resolved by a change in the brain-body-environment system. These gradients are the result of the individual's selective openness to the available affordances which is the result of dynamical patterns evolving at multiple time-scales. The dynamics unfolding over long timescales act as control parameters or constraints for dynamics unfolding over shorter timescales. Crucially, when the dynamical system (generative model) and the environmental dynamics are well attuned to each other, the solicitations/gradients/prediction errors that stand out as to-be-responded-to are the ones that lead toward an optimal grip on the environment.

An open question that remains is the following: what does it mean to say, under the FEP, that the organism and the environment are well attuned to each other? In other words, what aspects of the environment must the generative model be reflecting for the organism to interact adequately with its environment? We will address these questions in the next section.

## Situating the anticipating brain

Radical Embodiment emphasizes the non-decomposability of the brain-body-environment system, which implies that the neural dynamics can only be studied while taking into account the larger brain-body-environment dynamics (Chemero, 2009). When focusing on one element of these dynamics, such as the brain, one can model the rest of the dynamics as control parameters (Friston, 2000). This allows for several perspectives on essentially the same dynamics: the state variables of the brain-body-environment system can be control parameters for the brain. From this perspective, it is possible to focus on the dynamics of the brain^18^: in this case, the body and the environment are described as control parameters (prediction errors) that are changing themselves. Given that the brain is situated within a robust brain-body-environment system, one can derive constraints on how the brain is coupled to the wider system. Following this analysis of the dynamical coupling, one ends up with the perspective of the FEP.

If aspects of our brain can be predicted from our environment, we need to understand which aspects of the environment are being reflected in brain dynamics. The fundamental idea of the FEP is that by being equipped with a generative model that reflects the hierarchical and temporal organization of the changing environment, organisms are able to remain attuned with the dynamics of the environment. This invites the question how the landscape of affordances, introduced in the first section of this paper, and the generative model/the organism are related to each other.

At several places Friston states that the agent is inferring the causal structure of the environment (e.g., Friston, 2011). However, it is important to qualify this in several respects. First, above, we have interpreted Friston's notion of inference in a non-propositional way fully within the domain of dynamical systems. Second, the agent is not modeling the causal structure of the environment *per se*, but rather those aspects of the environment that are important within its specific niche. We think that what is “inferred” in active inference, as we have noted above, are not objects or properties of objects, but rather anticipatory patterns that specify a solicitation. A pattern on which the system settles does not *represent*, say, a carrot, the smell of a carrot, or what to do with a carrot, but rather, the attractor state is directly coupled to the affordance of the carrot here and now (Freeman, 2000; Dreyfus, 2007): at no point in skillful action is the organism inferring the current causal state of the environment, and *on top of that* figuring out what change in the causal structure will lead to a more favorable outcome. Rather, the gradients/prediction errors themselves trigger the right anticipatory pattern that makes the right affordance stand out and that minimizes free energy or, in more phenomenological terms, leads to an optimal grip on the organism's environment.

Inspired by Gibson (1979) we have, as mentioned in the introduction, suggested that we can understand the ecological niche as a landscape of affordances (Kiverstein and Rietveld, 2012). Armed with our understanding of the richness of the landscape of affordances available in our form of life (as developed in the first part of the paper), we argue that what the embodied organism is “modeling” or reflecting in a particular situation, is not so much the causal structure of the environment *per se*, but rather the dynamic nested structure of the field of affordances. We do not think this is in contradiction with the FEP but rather a natural consequence of combining active inference (action and perception jointly reducing gradients/prediction errors) and the need for the organism to be governing its interactions with the environment.

This contextualization of the anticipating brain is important for two reasons. First, it makes clear that the FEP really calls for an integrative approach for understanding the mutual attunement of the brain and the other components of the whole brain-body-environment system. The deep correspondence between the dynamics in the environment and the neurodynamics implies that we can learn something about the brain by investigating the structure of the econiche, i.e., of the landscape of affordances.

Second, it provides a new understanding of the tendency toward an optimal grip, which is a central notion in phenomenology, as the concernful skilled agent's tendency to reduce his or her dis-attunement to the environmental dynamics. In particular, it provides an understanding of how the relevance of affordances is selectively brought: the relevance of an affordance (an attribute of the brain-body-environment system) is in part brought about by aspects of the environment triggering patterns that shape the skillful agent's action-readiness for interacting with its environment. We think that the field of affordances both captures an important aspect of the phenomenology of skilled intentionality, and can inform theoretical neuroscientists about what it is the self-organizing brain is responsive to (i.e., what external control parameters influence the self-organization of the brain). Skilled intentionality should be of particular interest to those who work on the implications of the free-energy principle, because it is the kind of intentionality manifested when we act as “surprisallessly” as possible: when we are in familiar environments and can act relatively unreflectively and effortlessly.

## Toward a radical embodied cognitive neuroscience

In the previous sections, we have presented an integrative framework for studying skilled intentionality. In this section we will illustrate the plausibility of our framework by presenting work on metastability in the system “brain-body-landscape of affordances” dynamics of skilled sportsmen, and empirical research on the impact of DBS on affordance responsiveness of OCD patients.

### Metastability and optimal grip

Above we have seen that metastable dynamics are an important characteristic of neurodynamics, because it allows for context-sensitive selective openness and flexible switching between activities. An interesting property of metastable dynamics in the brain, like the stable heteroclinic channels described in Section The Neurodynamics of Selective Openness for example, is the possibility to be both robust to perturbations and flexible^19^. The dynamics of the coupled patterns generators can be described as visiting a succession of unstable fixed points in an abstract state space (Tsuda, 2001; Rabinovich et al., 2008). The itinerant dynamics can be observed at different time-scales or at different levels of the hierarchy. One can see how such a system can be both robust and flexible: on the one hand do slower-evolving dynamics constrain the faster-evolving dynamics, on the other hand, because of the metastable character of the slower dynamics, some perturbations (e.g., as a result of gradients/prediction errors) can easily and swiftly change the slower dynamics and make it shift to a new pattern that better fits with the multiplicity of affordances currently encountered.

Importantly, metastable dynamics in the brain-body-environment system as a whole provide an important paradigm for understanding movement pattern variability in ecological situations. For example, Hristovski et al. (2006, 2009) investigated how boxers' striking patterns differed when manipulating the distance to a boxing bag. At great distances, they observed a “jab” movement, while at short distances, they observed “hooks” and “uppercuts.” At a critical distance of 0.6 (the distance to the punch bag scaled by the arm length), they found an optimal metastable performance region where a varied and creative range of movement patterns occurred: a region in which the boxers “could flexibly switch between any of the boxing action modes” (Chow et al., 2011, p. 197). So, at different scaled-body distances, the boxing bag solicited different punches, but at the optimal metastable distance, the boxing bag solicited a wide variety of punches. Here something occurs that might be called a *Hypergrip on the field of affordances* (Rietveld, 2013). For an expert boxer the zone of optimal metastable distance will solicit moving toward, because this zone offers a wide range of action opportunities and the possibility to flexibly switch between them in line with what the dynamically changing environment demands or solicits.

Anticipation is an important aspect of the phenomenon of Hypergrip on the field of affordances. This is best illustrated by means of an example from a different field of expertise. In ice-climbing, the metastable regime is one where the expert climber can use different movement patterns to obtain the same result (Seifert et al., 2014). Moreover, a skilled climber is anticipating the affordances ahead; she does not just get a grip on the next hold in climbing, say, but also anticipates that she needs to be able to move on after that. So, the question of relevance sensitivity is not just about grasping the next hold, but rather about which of the available holds afford obtaining a grip on the *whole climbing route ahead*. One can see again that in such a metastable state, one is flexibly able to switch between different movement regimes and better fit to adapt to the specific details of the environmental aspects.

These studies suggests that, at least in some domains of skilled action, we can formalize the tendency toward an optimal grip in terms of the occurrence of metastable movement patterns. More precisely, we can understand the tendency toward an optimal grip as the tendency toward an *optimal metastable attunement* to the dynamics of the environment. This optimal readiness to switch between behavioral patterns is both functional with respect to the demands of the environment and the needs of the organism.

Further empirical research on optimal metastable performance regions in ecological psychology will thus be able to illuminate the phenomenon of the tendency toward an optimal grip and the selective openness to relevant affordances. It will be particularly interesting to see what agents will do in situations in which there is not a specific task given, or when they are allowed to switch spontaneously between different ways to solve a task, just like in everyday life.

Moreover, the phenomena of flexible switching and Hypergrip on the field of affordances on the horizon touch upon one of the most important open questions in cognitive science, the frame problem (Wheeler, 2008; Rietveld, 2012b). Skilled intentionality treats context as just more affordances—a landscape of affordances available in an ecological niche—and avoids the frame problem by starting from the phenomenon of maintaining grip on multiple affordances simultaneously.

How can the neurodynamics involved in selective openness support an optimal grip on the whole field of affordances including possibilities for action on the horizon? In order to answer this question, we need to understand how the self-organized metastability of the brain-body-environment system interacts with the self-organized metastability of the brain. To advance, it is important to develop neuroscientific research methods that are able to complement the work done on boxing and climbing in an actual ecological setting. What is the difference in neurodynamics in the optimal metastable region as compared to the other regions of performance? In the next section, we present recent research by our group on the impact of DBS on affordance responsiveness as an example of such a complementary approach that also takes phenomenology seriously.

### Mood disorders and relevance-sensitivity

Our understanding of relevance sensitivity as the self-organized coordination of action-readiness patterns is closely related to Frijda's theory of emotions (Frijda, 1986, 2007). According to his theory, the key aspect of an emotion is a state of action-readiness for changing an aspect of the self-object relationship.

**Figure 2:**
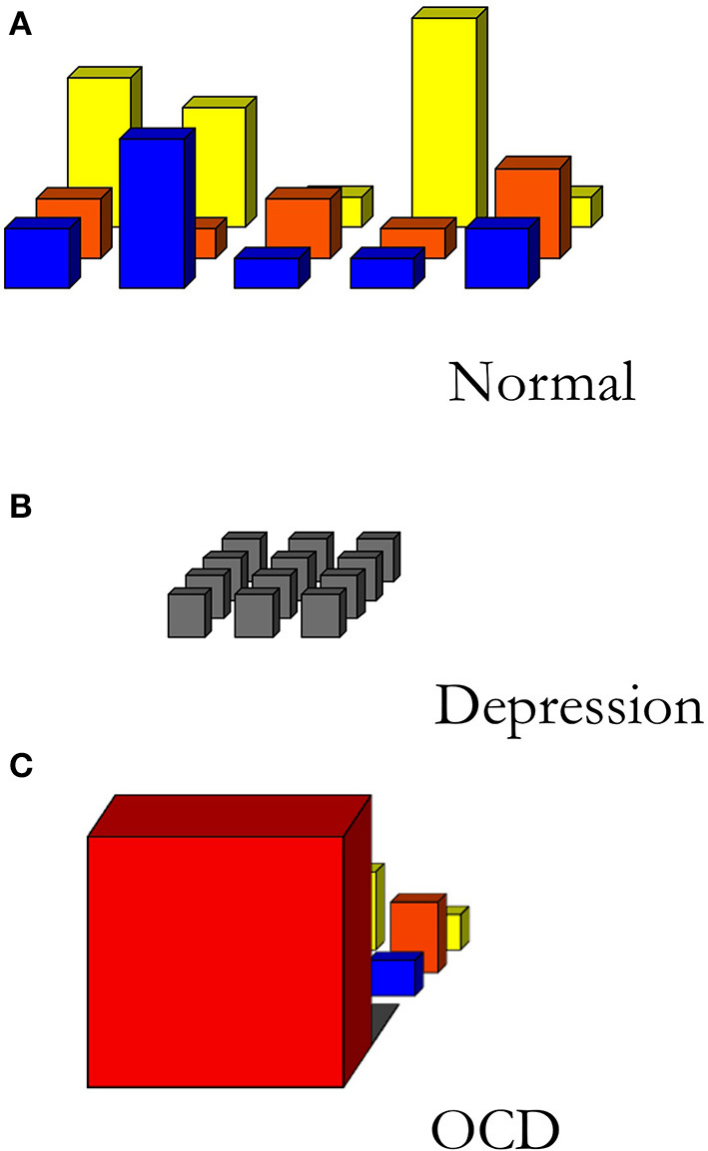
**Sketch of different fields of relevant affordances**. **(A)** A normally structured, differentiated field of affordances. **(B)** The field of affordances of a depressed person. **(C)** The field of affordances of an OCD-patient. Creative Commons license applies (de Haan et al., 2013).

Moods are action-readiness patterns that persist for longer periods of time and typically have a relatively global character: a mood is reflected in the structure of field of affordances as a whole (See Figure 2A). Similarly, we can understand mood disorders as disorders that distort the field of affordances: in the case of depression, for example, the field of affordances is rather flat, nothing stands out as attractive or soliciting anymore (see Figure 2B). A recent qualitative study investigates the effects of DBS on the phenomenology of patients suffering from treatment resistant OCD (de Haan et al., 2013). OCD can be characterized by the presence of anxiety-provoking thoughts, typically followed by ritualistic behaviors (compulsions) to relieve the anxiety. In extreme cases of OCD, the patient's field of affordances is narrowed down to just the immediate solicitation of what has to be done here and now without the possibility of flexibly switching to a new readiness for other behaviors (See Figure 2C).

DBS treatment consists of permanently implanted electrodes that deliver electrical pulses to a target brain region. DBS of the nucleus accumbens shows promising results as treatment for OCD patients (Denys et al., 2010). It is hypothesized that, rather than merely having inhibitory or excitatory effects on the target area, DBS restores intrinsic brain network dynamics (Figee et al., 2013). In particular, the authors show that DBS treatment normalizes the activity of the nucleus accumbens and restores the intrinsic frontostriatal network dynamics. These frontostriatal circuits are known to be important for switching between different actions (Ridderinkhof et al., 2011). Furthermore, it was found that the frontostriatal connectivity changes strongly correlated with OCD symptom improvement (Figee et al., 2013).

From phenomenological interviews with these OCD-patients, it becomes clear that, by treatment with DBS combined with cognitive behavioral therapy, these patients report a general change in engagement with the world pertaining to perception, reflection, mood, interests and social interaction (Rietveld et al., 2013). These impressive phenomenological changes can be understood as changes in the responsiveness to the field of affordances along three dimensions: the “width” (the scope of affordances engaged with), the “depth” (the temporal horizon), and the “height” (the relevance) of the field of affordances (see Figure 2) (de Haan et al., 2013).

Above we have argued that affordance-responsiveness corresponds to heavily interacting neurodynamics at different temporal scales. DBS can directly perturb these neurodynamics and hence directly influence the general capability of affordance-responsiveness (de Haan et al., 2013). The observed change in affordance-responsiveness opens up the possibility to develop neurodynamical models of OCD and psychiatric disorders more generally, based on Friston's ideas on the anticipating brain. Friston's model of addiction (2012c) highlights the importance of metastable, itinerant dynamics for modeling adaptive and pathological behavior. In personal communication (June 15th, 2012) Friston has suggested that OCD could also be modeled along the same lines. We are currently working on a research project that uses our framework and an updated version of Friston's (2012c) model for addiction, to generate testable hypotheses about the neural mechanisms that could underlie the breakdown of normal affordance-responsiveness in OCD-patients and its recovery through DBS treatment.

## Conclusion

In this paper, we have investigated the phenomenon of skilled intentionality from the perspective of the self-organization of both the brain-body-environment system and the brain. Previously, we have characterized skilled intentionality as the organism's tendency toward an optimal grip on a field of relevant affordances. In this paper we have investigated the mechanisms that underlie the self-organized selective openness to available affordance and the organism's tendency toward an optimal grip. We have integrated different perspectives on this phenomenon: the philosophy of skilled of intentionality, Kelso's and Tschacher and Haken's ideas on self-organization, Friston's theory of the anticipating brain, and work on metastable dynamics. What these four perspectives on the affordance-responsive active individual have in common is the idea that a an organism self-organizes by reducing a disequilibrium in the brain-body-environment system. On the different levels of analysis, this disequilibrium can be called a solicitation, a gradient or a prediction error, or a dis-attunedness of internal dynamics and environmental dynamics.

Our integrated framework moves beyond the traditional Gibsonian conception of affordances, because it highlights that the animal cares about certain things and needs to be selectively open to the relevant affordances in a particular situation. Explaining this selective openness to affordances in dynamical terms should be the main focus for Radical Embodied Cognitive Neuroscience. Friston's neurodynamical models provide an interesting perspective on the possible neural mechanisms that underlie the selective openness to relevant affordances. Furthermore, the tendency toward an optimal grip could, within the perspective provided by the FEP, be seen as a consequence of the continuous attunement of the internal dynamics and external dynamics through affordance-responsiveness. We have suggested that the situated anticipation of an affordance generates an action-readiness pattern that makes the affordance stand out as relevant.

Although the FEP alludes to the attunement of the dynamics in the environment and the organism's own dynamics, the body of work in ecological psychology on the rich metastable dynamics in the brain-body-environment system that often is overlooked by the people working on neurodynamics. We have suggested that by bringing together these two approaches with our phenomenology of skilled action, it is possible to develop an integrative research project for understanding affordance responsiveness in both healthy and pathological cases.

From the picture presented in this paper it becomes clear that the common ground for researchers in radical embodied cognitive science, ecological psychologists and dynamical computational neuroscientists is the view that the brain is an intrinsically instable dynamical system embedded in the broader system “brain-body-landscape of affordances.” The central way in which an organism relates to its environment is by the organism as a whole tending toward an optimal grip on the field of affordances. It is this phenomenon that should be a central topic in Radical Embodied Cognitive Neuroscience.

In his influential article on action-oriented predictive processing, Clark (2013) is hesitant to embrace the more radical implications of the FEP. In Friston's full Free Energy story, one does not need to appeal to desires, goals and rewards in order to explain behavior, but one can replace them with prediction and anticipation; utility functions are replaced by the minimization of prediction error. Clark dubs this resulting picture the “desert landscape” scenario.

However, we think this scenario is rather appealing because unlike almost all work in cognitive neuroscience it does not presuppose the presence of a “goal” or “desire” of which it is completely unclear how it was selected out of the many possible “goals” or “desires.” Furthermore, the title “desert landscape” is a misleading depiction of the human ecological niche or landscape of affordances, which is in fact very rich independently of any particular individual. Moreover, we have seen that the skilled individual does not have an explicit goal in mind, but rather is solicited or invited by the field of affordances. We think that what is at the root of skilled activity is not a set of desires or goals, but rather the ongoing modulation of coupled self-organizing dynamical systems that results in the adequate interaction of an organism with its environment.

The best way to characterize these dynamics is in terms of anticipation and attunement. This radical version of the FEP should appeal to enactivists and embodied cognitive scientists, for it does not posit an explanatory role for propositional internal states at the basis of our cognitive system and takes self-organization seriously. Clark's main objection against the desert landscape scenario is that, even if it were true, it will not provide the best way of making sense of the rich organization of our cognitive organization. We agree with Clark here. As we have shown in this paper, an appeal to anticipation and attunement on the sub-personal level in no sense precludes us from highlighting the rich phenomenology of skilled action and the valuable affordances available in the ecological niche. The resulting picture then is not, as it is according to Clark, a barren desert landscape, but rather one of engagement with a flourishing field of affordances.

### Conflict of interest statement

The authors declare that the research was conducted in the absence of any commercial or financial relationships that could be construed as a potential conflict of interest.

## Footnotes

^1^The word grip has several connotations in the English language. It can refer to a physical grip (such as when grasping a cup), but also a more intellectual grip (such as when having a grip on a problem), as well as a grip in the sense of being able to deal with something (such as when losing your grip on a situation). As we state in the text, skilled action pertains to simple motor behaviors, but also to more complex and context-sensitive actions. Optimal grip is, because of the multiple connotations of the word grip, supposed to characterize all these aspects of the phenomenology of skilled action.

^2^This is a remark that concerns the structure of the ecological niche and not our phenomenology. Phenomenologically, the structure of our experience of solicitations resembles more that of a field with some solicitations standing out and with a horizon. Moreover, this is not to deny the fact that our lives proceed along paths or trails, as Ingold (2011, p. 147) rightfully stresses, “always on the way from one place to another.”

^3^We do not wish to claim that the landscape has a clear hierarchical structure. Rather, the structure would be more like a heterarchy. That is to say, there is no strict demarcation of levels within the nested structure, although when focusing on a specific event, such as dining in a restaurant, place-affordances can be discerned.

^4^These are all standard definitions, in this case obtained from Chemero (2009), Kelso (2012), Rabinovich et al. (2008), Tschacher (2010).

^5^The notion of gradient has a clear physical interpretation in the case of the Bénard effect: it is the difference in temperature between the top and the bottom of the layer of fluid. In the case of the coordination dynamics of locomotion, the gradient is for instance the speed of the treadmill to which the animal adapts its gait. Tschacher and Haken (2007) give an example of a psychological gradient guiding an action: in the context of a letter that one has to mail, the letter-affording-delivery stands out as a gradient to be reduced.

^6^Tschacher and Haken (2007) do not make the distinction between solicitations and affordances. Their use of the word “affordances” applies to gradients that actually drive the system (i.e., what we call solicitations).

^7^An important open question is that of optimality: on some interpretations of self-organization (Schneider and Kay, 1994), the pattern that arises is always the one that most efficiently (i.e., in the least amount of time) dissipates the gradient. As Haken and Tschacher (2010) point out, it is not clear that such an optimality principle for self-organizing systems in general is feasible.

^8^Iberall writes: “[Self-serving systems] can explore its surround to acquire the necessary potentials at its boundary that serve as sources of free energy for its own internal and externalized processes. In this case internal processes convert internal energy into a useful form of work that can change momentum and move the system to a favorable location (1977, p. 177).”

^9^What follows is a treatment of the theory of the FEP. For mathematical details, see Friston (2006, 2012b).

^10^Because under ergodic assumptions, entropy is equal to the average of self-information (surprisal), see Friston et al. (2009) for mathematical details.

^11^The latter has a clear physical definition in terms of the amount of energy available in a system that is convertible to work. The former is a quantity from information theory, which is an upper bound on surprisal. As such, information theoretic free energy has nothing to do with energy in the ordinary sense of the word.

^12^Standard definitions taken from Friston (2010).

^13^Within the context of the FEP, much attention in the literature has been given to how efficient information processing is possible (in the form of predictive-coding and approximate Bayesian inference), however, much less attention has been given to what structures in the environment the anticipating organism is responsive to. In this paper we are concerned with the latter question.

^14^Although the FEP uses the word “model,” we think that it is used in a way that makes it sufficiently compatible with radical embodiment. What radical embodiment is against, is the idea that an agent has an internal model of the world, which, through some inference process, provides the agent with a representation of the world on which it consequently can decide what to do. This is not what the FEP entails.

^15^The radical response would be to question the added explanatory value of the notion of inference over and above the dynamical explanation (Chemero, 2009). We lack the space here to retranslate the FEP in non-propositional, dynamical terms, but we think that this is possible. For the moment, it is important to emphasize that notions such as “inference,” “belief,” and “expectation” all have a different meaning within computational neuroscience and philosophy.

^16^In fact, Friston uses the word “affordance” to designate the activation patterns that guide affordance responsiveness. This is not in line with how the term is traditionally used in ecological psychology (Gibson, 1979) and philosophy (Chemero, 2003) and is bound to lead to confusion. We will use “action-readiness pattern” to designate what Friston calls “affordance.”

^17^An intuitive example of a stable heteroclinic channel would be a pub-crawl. One visits a sequence of bars (the metastable saddle points), while walking from one to another in between (the transients). The sequence might be fixed, but the timing for when to move to a new bar is generally left to the specific circumstances.

^18^Note that this pertains to the domain of coupled dynamical systems. Given the centrality of the brain-body-environment system as a whole, we do not think the possibility of constructing such a perspective from the brain justifies epistemic internalism.

^19^This contrasts with phase locked dynamics. Phase locked dynamics are generally robust to small perturbations as well, but lack the flexibility of metastable dynamics.
